# Post-surgical outcomes of patients with chronic kidney disease and end stage renal disease undergoing radical prostatectomy: 10-year results from the US National Inpatient Sample

**DOI:** 10.1186/s12882-019-1455-2

**Published:** 2019-07-23

**Authors:** Chen Ning, Xinyi Hu, Fangming Liu, Jun Lin, Jian Zhang, Zhipeng Wang, Yichen Zhu

**Affiliations:** 1 Department of Urology, Capital Medical University Beijing Friendship Hospital, No.95 Yong’an Road, Xicheng District, Beijing, 100050 China; 2 Beijing Key Laboratory of Tolerance Induction and Organ Protection in Transplantation, No.95 Yong’an Road, Xicheng District, Beijing, 100050 China

**Keywords:** Chronic kidney disease (CKD), End-stage renal disease (ESRD), Prostate cancer, Radical prostatectomy, Robot-assisted, National Inpatient Sample (NIS)

## Abstract

**Background:**

Chronic kidney disease (CKD) and end stage renal disease (ESRD) are not well characterized in prostate cancer patients. This study aimed to examine the clinical characteristics and postsurgical outcomes of patients with or without CKD and ESRD undergoing radical prostatectomy for prostate cancer.

**Methods:**

This population-based, retrospective study used patient data from the Nationwide Inpatient Sample, the largest all-payer US inpatient care database. From 2005 to 2014, 136,790 male patients aged > 20 years diagnosed with prostate cancer and who received radical prostatectomy were included. Postoperative complications, postoperative acute kidney injury (AKI) and urinary complications, and length of hospital stay were compared between patients with or without underlying CKD and ESRD.

**Results:**

After adjusting for relevant factors, the CKD group had a significantly higher risk of postoperative complications than the non-CKD group. In addition, the CKD group had a 5-times greater risk of postoperative AKI and urinary complications than the non-CKD group. Both CKD and ESRD groups had significantly longer hospital stays than the non-CKD group. Patients receiving RARP had a lower risk of postoperative complications than those who received open radical prostatectomy, regardless of having CKD or not. Both non-CKD and CKD patients receiving RARP had shorter hospital stays than those who received open surgery.

**Conclusions:**

Prostate cancer patients with underlying CKD had significantly greater risk of postoperative complications, postoperative AKI and urinary complications, and longer hospital stays than those without CKD. The use of RARP significantly shortened hospital stays and reduced complications for these patients.

**Electronic supplementary material:**

The online version of this article (10.1186/s12882-019-1455-2) contains supplementary material, which is available to authorized users.

## Background

Acute kidney injury (AKI), estimated to occur in 21% of all hospital admissions worldwide, is associated with increased disease burden, healthcare costs, and mortality [1]. In addition to the higher risk for renal replacement therapies (RRTs) such as dialysis and kidney transplant, such patients also are at higher risk for cardiovascular events, fractures, and anemia [2]. Further, hospitalized patients with AKI are at 8–9 times greater risk of developing chronic kidney disease (CKD) and 3 times greater risk of developing end-stage renal disease (ESRD) [1]. A 2017 World Bank report estimated that the prevalence of CKD, defined in 2002 as an estimated glomerular filtration rate (eGFR) < 60 mL/min/1.73 m^2^ [3], is likely to be similar in developed and developing countries, although the etiologies and populations will differ. Estimates of prevalence range from 7 to 15%. Prevalence of ESRD is shown to be much lower, about 0.03%. While Western CKD and ESRD patients are often older adults with diabetes and hypertension, those in developing countries are younger, and reduced kidney function is associated with infectious diseases (e.g., HIV/AIDS, malaria, leptospirosis), herbal medicines, obstetric complications, and exposure to environmental toxins [2]. The younger patients in developing countries also have much lower access to treatment; one study found that only 5% of ESRD patients in China, India, and Nigeria have access to RRT [4].

Whatever the etiology, CKD is a significant risk factor for increased post-surgical morbidity, which includes longer hospital stays, particularly in men receiving radical prostatectomy [5–7]. One study on the impact of CKD on early postoperative outcomes in patients undergoing urological oncological surgery (including radical prostatectomy) concluded that renal dysfunction may be under-recognized in such patients, and CKD stages III, IV and V are independent predictors for poor 30-day postoperative outcomes [8].

Radical prostatectomy is the gold standard treatment for locally advanced prostate cancer, the most common type of cancer in men in the US [9, 10]. Advances in detection (including prostate specific antigen [PSA] levels, magnetic resonance imaging [MRI] and a refined Gleason score) have increased the number of men presenting with localized prostate cancer [9]. A Cochrane systematic review found few clinically relevant differences in outcomes for such patients between open radical prostatectomy (ORP) and either laproscopic radical prostatectomy (LRP) or robot-assisted radical prostatectomy (RARP).

Prostate cancer-specific survival has not been addressed in controlled trials that directly compared laparoscopic radical prostatectomy and RARP with ORP [11]. Also, data regarding the clinical features and postoperative outcomes of patients who received a radical prostatectomy with CKD and ESRD are limited. Therefore, we used the comprehensive National Inpatient Sample (NIS) database from 2005 to 2014 to clarify: 1) the characteristics and 2) in-hospital outcomes of patients undergoing radical prostatectomy for prostate cancer with or without CKD or ESRD. We hypothesized that patients with baseline CKD and ESRD who underwent radical prostatectomy for prostate cancer would have higher rates of postoperative morbidities and longer hospital stays than those with intact kidney function. In addition, we also evaluated whether RARP leads to better postsurgical outcomes than open surgery in all cases and in subgroups according to kidney health status.

## Methods

### Data source

In this population-based, retrospective observational study, we used the NIS database, the largest all-payer, continuous US inpatient care database that includes about 8 million hospital stays each year [12]. The data elements include primary and secondary diagnoses, primary and secondary procedures, admission and discharge status, patient demographics, expected payment source, length of stay, and hospital characteristics. All patients are considered for inclusion. The most recent NIS database contains data from about 1,050 hospitals from 44 States in the US, sampled to approximate a 20% stratified sample of US community hospitals as defined by the American Hospital Association.

### Study population

The primary cohort included male adults ≥20 years old with prostate cancer in the US as identified in the NIS database between 2005 and 2014, with an International Classification of Diseases, Ninth Revision (ICD-9) diagnosis code of 185.0, and receipt of radical prostatectomy (ICD-9: 60.5). The study cohort was further stratified by CKD status into the following groups: non-CKD; not dialysis-dependent CKD (ICD-9: 585.1–585.5, 585.9); and ESRD (ICD-9: 585.6 or with procedure code for hemodialysis or peritoneal dialysis: 39.95, 54.98, except when dialysis was performed for AKI: 584.5 to 584.9). This approach has been used to accurately identify patients in the NIS database with CKD or ESRD [13, 14].

### Variables

The primary endpoints were postoperative complications, postoperative AKI and urinary complications, and length of hospital stay. Postoperative complications were defined using ICD-9 codes and Clinical Classifications Software (CCS) codes. CCS is a tool developed at the Agency for Healthcare Research and Quality (AHRQ) for clustering patient diagnoses and procedures into clinically meaningful categories [15]. The following codes were used: cardiovascular complications: 997.1, 997.02, 997.09, 998.0, 100CCS; bleeding complications: 285.1, 998.1–998.2; pulmonary complications and pneumonia: 518.5, 518.81, 997.3, 122CCS; infection/sepsis: 998.5, 995.9; deep vein thrombosis (DVT)/ pulmonary embolism: 451.11, 451.19, 451.2, 451.81–84, 451.89, 451.9, 453.40–42, 453.8, 453.9, 997.2; wound complications: 998.12–998.13, 998.3, 998.5; device complications: 996.1, 996.62, 996.74; and other complications: 997.0, 997.4, 997.6–997.9, 998.2, 998.4, 998.6–998.9. Postoperative AKI and urinary complications were identified using ICD-9 codes 584, 997.5, 157CCS. Because the billing codes contained mixed diagnoses of AKI and other urinary complications that could not be separated, it was necessary to refer to a composite outcome.

Patient characteristics extracted included age, race, income, insurance status (primary payer), and surgical approach. RARP was defined by ICD-9 procedure code 17.4. Comorbidities were identified from the database using algorithms validated by Elixhauser et al. [16]. Hospital-related characteristics (bed size, location, teaching status, hospital region and annual caseload of radical prostatectomy) were extracted from the NIS database as part of the comprehensive data available for all cases.

### Statistical analysis

Continuous variables are presented as mean with standard error (SE) and tested by ANOVA. Categorical variables are presented as weighted percentages and tested by Chi-square test. Logistic regression analyses and linear regression analyses were conducted to evaluate associations between the extent of kidney disease and clinical outcomes (postoperative complications, postoperative AKI and urinary complications, and length of stay). The variables that were significantly associated with the extent of kidney disease at baseline were included in multivariate regression models after adjusting for confounders. Complete case analysis was used in multivariate models. Regression models were stratified by the extent of kidney disease to evaluate associations between clinical outcomes and surgical approach (RARP versus ORP). Discharge weights were applied to the mean, SE, proportions, all testing, and regression models to account for the NIS sampling method. A 2-sided *p* value of < 0.05 was considered statistically significant. Statistical analyses were performed using the SAS statistical software package, version 9.4 (SAS Institute Inc., Cary, NC, USA).

## Results

### Study population

**Fig. 1:**
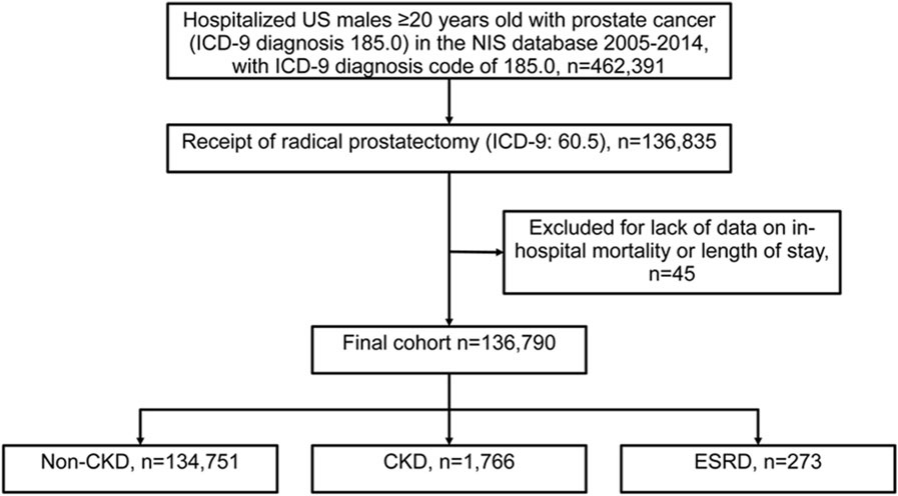
Study sample selection. NIS, Nationwide Inpatient Sample; ICD-9, International Classification of Diseases, Ninth Revision; CKD, chronic kidney disease; ESRD, end-stage renal disease

Selection of the study cohort is described in Fig. 1. In 2005–2014, 462,391 male patients aged > 20 years were diagnosed with prostate cancer. Among these, 136,835 patients received radical prostatectomy. After excluding 45 cases without data for postoperative mortality or length of stay, the data of 136,790 patients were available for subsequent analyses. Patients’ demographics, hospital characteristics and clinical outcomes are summarized in Tables 1 and 2. Patients with differences in the extent of kidney disease were found to have significant differences in surgical approach, age, race, income by ZIP code, insurance status, location and teaching status of hospital, region of hospital, hospital caseload of radical prostatectomy, Elixhauser comorbidity score, and comorbidities, including anemia, congestive heart failure, chronic pulmonary disease, coagulopathy, depression, diabetes, hypertension, fluid/electrolyte disorders, obesity, peripheral vascular disorders and weight loss (all *p* ≤ 0.02) (Table 1). Patients with either CKD or ESRD had longer hospital stays, greater likelihood of postoperative complications, and postoperative AKI and urinary complications than did the non-CKD group (all *p* ≤ 0.001) (Table 2).

**Table 1:** Descriptive statistics of surgical approaches, demographic data, and hospital characteristics by extent of kidney disease

Variables	All patients (*N* = 136,790)	Non-CKD (*n* = 134,751)	CKD (n = 1,766)	ESRD (*n* = 273)	*p*-value^1^
Surgical approach					<.001
Robot-assisted	58703 (43)	57614 (42.8)	957 (54.1)	132 (48.5)	
Open	78087 (57)	77137 (57.2)	809 (45.9)	141 (51.5)	
Demographic data					
Age	61.37 ± 0.05	61.34 ± 0.05	64.5 ± 0.18	60.34 ± 0.4	<.001
Race					<.001
Missing	22526 (16.4)	22230 (16.5)	265 (14.9)	31 (11.3)	
White	87803 (64.3)	86795 (64.5)	904 (51.3)	104 (38.2)	
Black	13597 (9.9)	13078 (9.7)	423 (23.9)	96 (35.3)	
Hispanic	6802 (5)	6685 (4.9)	92 (5.2)	25 (9)	
Other	6062 (4.4)	5963 (4.4)	82 (4.6)	17 (6.2)	
Income					<.001
Missing	3506 (2.6)	3469 (2.6)	32 (1.8)	5 (1.9)	
0-25th percentile	24745 (18)	24257 (18)	408 (23.2)	80 (29.4)	
26th to 50th percentile	30884 (22.5)	30363 (22.5)	447 (25.4)	74 (27.1)	
51st to 75th percentile	35469 (25.9)	34987 (26)	422 (23.9)	60 (21.8)	
76th to 100th percentile	42186 (31)	41675 (31)	457 (25.7)	54 (19.8)	
Insurance status					<.001
Missing	256 (0.2)	254 (0.2)	1 (0.1)	1 (0.4)	
Medicare/Medicaid	45803 (33.5)	44683 (33.1)	938 (53.2)	182 (66.3)	
Private/HMO	84938 (62.1)	84089 (62.4)	765 (43.2)	84 (31.1)	
Self-pay/no-charge/other	5793 (4.2)	5725 (4.2)	62 (3.5)	6 (2.2)	
Hospital characteristics					
Bed size					.27
Missing	720 (0.5)	703 (0.5)	15 (0.8)	2 (0.8)	
Small	14495 (10.3)	14280 (10.3)	191 (10.4)	24 (8.3)	
Medium	28040 (20.7)	27590 (20.67)	397 (22.9)	53 (19.7)	
Large	93535 (68.5)	92178 (68.5)	1163 (65.9)	194 (71.3)	
Location and teaching status					.02
Missing	720 (0.5)	703 (0.5)	15 (0.8)	2 (0.8)	
Rural	6294 (4.6)	6203 (4.6)	84 (4.7)	7 (2.5)	
Urban nonteaching	40144 (29.1)	39571 (29.1)	518 (29.3)	55 (19.6)	
Urban teaching	89632 (65.9)	88274 (65.8)	1149 (65.2)	209 (77.2)	
Region					<.001
Northeast	25341 (19.2)	25046 (19.3)	237 (13.9)	58 (22.1)	
Midwest	32953 (24.4)	32348 (24.3)	543 (30.9)	62 (22.5)	
South	48141 (34.7)	47480 (34.7)	567 (31.8)	94 (34)	
West	30355 (21.8)	29877 (21.7)	419 (23.4)	59 (21.4)	
Radical prostatectomy caseload^a^					<.001
0-25th percentile	32941 (24.1)	32264 (24)	606 (34.6)	71 (25.5)	
26th to 50th percentile	35214 (25.7)	34646 (25.7)	491 (27.8)	77 (28.1)	
51st to 75th percentile	34151 (24.8)	33741 (24.9)	347 (19.6)	63 (23.4)	
76th to 100th percentile	34484 (25.3)	34100 (25.4)	322 (18)	62 (23.1)	
Comorbidities					
Elixhauser comorbidity score^b^					<.001
0-25th percentile	48823 (35.7)	48811 (36.2)	11 (0.6)	1 (0.4)	
26th to 50th percentile	50502 (36.9)	50366 (37.4)	131 (7.4)	5 (1.8)	
51st to 75th percentile	25410 (18.6)	24894 (18.5)	471 (26.6)	45 (16.4)	
76th to 100th percentile	12055 (8.8)	10680 (7.8)	1153 (65.3)	222 (81.4)	
Anemia	4774 (3.5)	4362 (3.2)	287 (16.2)	125 (45.8)	<.001
Congestive heart failure	820 (0.6)	718 (0.5)	82 (4.7)	20 (7.3)	<.001
Chronic pulmonary disease	10580 (7.7)	10310 (7.7)	241 (13.8)	29 (10.7)	<.001
Coagulopathy	879 (0.6)	815 (0.6)	56 (3.2)	8 (2.9)	<.001
Depression	5459 (4.0)	5353 (3.9)	93 (5.3)	13 (4.8)	.02
Diabetes	17106 (12.5)	16405 (12.2)	602 (33.9)	99 (36.4)	<.001
Hypertension	66931 (50.9)	65151 (48.3)	1521 (86.1)	259 (94.8)	<.001
Fluid/electrolyte disorders	3755 (2.8)	3449 (2.5)	254 (14.4)	52 (19.3)	<.001
Obesity	9411 (6.9)	9101 (6.8)	279 (15.7)	31 (11.5)	<.001
Peripheral vascular disorders	1465 (1.1)	1368 (1.0)	89 (5.0)	8 (3.1)	<.001
Weight loss	230 (0.2)	209 (0.2)	18 (1.0)	3 (1.0)	<.001

Continuous variables are presented as mean ± standard error

Categorical variables are presented as unweighted counts (weighted percentage)

Percentages may not add up due to missing values

*CKD* chronic kidney disease, *ESRD* end stage renal disease

^1^Chi-square *p*-values are given for categorical variables, and Wald F-test p-values for continuous variables

^a^Hospital annual radical prostatectomy caseload was defined using quartiles (Q_1_ = 22, Q_2_ = 69, Q_3_ = 169)

^b^Elixhauser comorbidity score was defined using quartiles (Q_1_ = 0, Q_2_ = 1, Q_3_ = 2)

**Table 2:** Descriptive statistics of clinical outcomes by extent of kidney disease

Clinical outcomes	All patients (N = 136,790)	Non-CKD (*n* = 134,751)	CKD (n = 1,766)	ESRD (n = 273)	*p*-value^1^
Length of stay	2.11 ± 0.02	2.09 ± 0.02	3.16 ± 0.11	4.33 ± 0.41	<.001
Postoperative complication	12434 (9.1)	12011 (8.9)	364 (20.6)	59 (21.6)	<.001
In-hospital mortality	55 (0.04)	51 (0.04)	2 (0.1)	2 (0.7)	<.001
Cardiovascular complications	943 (0.7)	889 (0.7)	46 (2.7)	8 (3)	<.001
Bleeding complications	7974 (5.8)	7708 (5.7)	230 (13)	36 (13.2)	<.001
Pulmonary complications	1251 (0.9)	1158 (0.9)	75 (4.2)	18 (6.5)	<.001
Infection/sepsis	303 (0.2)	273 (0.2)	22 (1.2)	8 (3)	<.001
DVT / Pulmonary embolism	204 (0.2)	189 (0.1)	13 (0.8)	2 (0.7)	<.001
Wound complications	694 (0.5)	662 (0.5)	24 (1.3)	8 (2.9)	<.001
Device complications	28 (0.02)	20 (0.02)	1 (0.1)	7 (2.5)	<.001
Other complications	4689 (0.08)	4575 (3.4)	102 (5.8)	12 (4.4)	<.001
Postoperative AKI and urinary complications^a^	1999 (1.5)	1704 (1.3)	295 (16.4)	–	<.001

Continuous variables are presented as mean ± standard error

Categorical variables are presented as unweighted counts (weighted percentage)

*CKD* chronic kidney disease, *ESRD* end stage renal disease, *DVT* deep vein thrombosis, *AKI* acute kidney injury

^1^Chi-square *p*-values are given for categorical variables, and Wald F-test *p*-values for continuous variables

^a^The ESRD group was not included in the analysis of postoperative AKI and urinary complications

### Associations between clinical outcomes and kidney disease

The results of regression analyses are presented in Table 3 and given in detail in Additional file 1: Table S1. The results showed that patients in the CKD and ESRD groups were at greater risk of postoperative complications than those in the non-CKD group (OR = 2.66 and 2.83, 95% CI = 2.36–3.00 and 2.14–3.75, respectively) (Table 3). However, after adjusting for age, race, income, insurance status, comorbidities, region, surgical approach, hospital location, teaching status, and radical prostatectomy caseload, only the CKD group had significantly higher odds of postoperative complications (adjusted odds ratio [aOR] = 1.36, 95% CI = 1.18–1.56). The CKD group had a 15.63-times higher risk of postoperative AKI and urinary complications than the non-CKD group (95% CI = 13.53–18.07); these results remained significant after adjustments (aOR = 5.16, 95% CI = 4.32–6.17). The CKD and ESRD groups had significantly longer hospital stays than the non-CKD group (β = 1.07 and 2.24, respectively), and these results also remained significant after adjustments (β = 0.25 and 0.98, respectively) (Table 3).

**Table 3:** Associations between clinical outcomes and extent of kidney disease

	Postoperative complication			Postoperative AKI and urinary complications^b^			Length of stay	
	n (%)	OR (95% CI)	aOR (95%CI)^a^	n (%)	OR (95% CI)	aOR (95% CI)^a^	β ± SE	β ± SE^a^
Non-CKD (n = 134,751)	12,011 (8.9)	Reference	Reference	1,704 (1.3)	Reference	Reference	Reference	Reference
CKD (n = 1,766)	364 (20.6)	**2.66 (2.36–3.00)**	**1.36 (1.18–1.56)**	295 (16.4)	**15.63 (13.53–18.07)**	**5.16 (4.32–6.17)**	**1.07 ± 0.11**	**0.25 ± 0.1**
ESRD (n = 273)	59 (21.6)	**2.83 (2.14–23.75)**	1.35 (0.97–1.87)	–	–	–	**2.24 ± 0.41**	**0.98 ± 0.4**

Significant values are in bold

*AKI* acute kidney injury, *OR* odds ratio, *CI* confidence interval, *aOR* adjusted odds ratio, *SE* standard error, *β* beta-coefficient, *CKD* chronic kidney disease, *ESRD* end-stage renal disease

^a^Multivariate analyses were adjusted for significant baseline characteristics, including surgical approach, age, race, income, insurance status, hospital location and teaching status, region, radical prostatectomy caseload, and all comorbidities

^b^The ESRD group was not included in the model of postoperative AKI and urinary complications

### Associations between clinical outcomes and type of surgery

We also conducted kidney disease-stratified regression analyses to compare types of surgery (RARP versus ORP) as shown in Table 4. Patients receiving RARP had lower risk of postoperative complications than those who received ORP, after adjusting for relevant factors [all patients: aOR (95% CI) = 0.55 (0.51–0.60); non-CKD: aOR (95% CI) = 0.54 (0.50–0.60); CKD: aOR (95% CI) = 0.65 (0.50–0.85); ESRD: aOR (95% CI) = 0.53 (0.21–0.91)] (Table 4). In the CKD group, those receiving RARP had lower risk of postoperative AKI and urinary complications than those receiving open surgery (OR = 0.75, 95% CI = 0.57–0.98); however, the results did not remain significant after adjustments (Table 4). After adjustments, non-CKD and CKD patients receiving RARP had shorter hospital stays than those who received open surgery (all patients: β = − 0.75; non-CKD: β = − 0.75; CKD: β = − 0.95) (Table 4).

**Table 4:** Association between surgical approach and clinical outcomes by extent of kidney disease

	Surgical approach	Postoperative complication			Postoperative AKI and urinary complications^b^			Length of stay	
		n (%)	OR (95% CI)	aOR (95% CI)^a^	n (%)	OR (95% CI)	aOR (95% CI)^a^	β ± SE	β ± SE^a^
All patients (N = 136,790)	Open	8,665 (6.3)	Reference	Reference	1,140 (0.8)	Reference	Reference	Reference	Reference
	Robot-assisted	3,769 (2.8)	**0.55 (0.50–0.60)**	**0.55 (0.51–0.60)**	859 (0.6)	1.01 (0.90–1.13)	1.09 (0.98–1.23)	**−0.77 ± 0.04**	**−0.75 ± 0.03**
Non-CKD (*n* = 134,751)	Open	8,432 (6.3)	Reference	Reference	987 (0.7)	Reference	Reference	Reference	Reference
	Robot-assisted	3,579 (2.7)	**0.54 (0.49–0.59)**	**0.54 (0.50–0.60)**	717 (0.5)	0.98 (0.87–1.09)	1.08 (0.96–1.22)	**−0.78 ± 0.04**	**−0.75 ± 0.03**
CKD n = 1,766)	Open	197 (11.2)	Reference	Reference	153 (8.7)	Reference	Reference	Reference	Reference
	Robot-assisted	167 (9.5)	**0.66 (0.52–0.84)**	**0.65 (0.50–0.85)**	142 (8.0)	**0.75 (0.57–0.98)**	0.83 (0.62–1.12)	**−1.32 ± 0.24**	**−0.95 ± 0.18**
ESRD (n = 273)	Open	36 (13.2)	Reference	Reference		–	–	Reference	Reference
	Robot-assisted	23 (8.4)	0.61 (0.34–1.09)	**0.43 (0.21–0.91)**				−0.34 ± 0.84	−0.66 ± 0.71

Significant values are in bold

*AKI* acute kidney injury, *OR* odds ratio, *aOR* adjusted odds ratio, *CI* confidence interval, *SE* standard error, *β* beta-coefficient; *CKD* chronic kidney disease, *ESRD* end stage renal disease

^a^Multivariate analyses were adjusted for significant baseline characteristics, including age, race, income, insurance status, hospital location and teaching status, region, radical prostatectomy caseload, and all comorbidities

^b^The ESRD group was not included in the model of postoperative AKI and urinary complications

## Discussion

In the present study, we examined the clinical characteristics and postsurgical outcomes of patients with or without CKD and ESRD undergoing radical prostatectomy for prostate cancer. We found that patients with underlying CKD had significantly increased risk of postoperative complications, including more than 5-times the risk of postoperative AKI and urinary complications than those without CKD. CKD patients also had longer hospital stays. In addition, comparison of outcomes by type of surgery was performed to further assess the possible benefit of robotic surgery. Those who received RARP had significantly lower risk of postoperative complications than those who received ORP, regardless of kidney health status. The use of RARP was associated with significantly shortened hospital length of stay only for CKD and non-CKD patients.

Other authors have compared postoperative outcomes in patients with CKD undergoing urological oncological surgery. In one study, which included 8,610 men receiving radical prostatectomy, 3,330 (38.7%) had no CDK and 5,280 (61.3%) had some level of CDK (ESRD not included). In the overall cohort, CKD was associated with increased odds of postoperative complications and longer hospital stays (*p* < 0.05), although the risk of complications was lower in prostate cancer patients than in those receiving other types of surgery (e.g., partial or radical nephrectomy or open radical cystectomy). Those authors noted that the prostate cancer patients, 65.4% of the total cohort, were healthier than the other types of patients included [8]. We also found that CKD was associated with significantly more postoperative complications and longer hospital stays relative to those with intact kidney function.

The risk of postoperative AKI after prostate surgery has been a subject of recent focus. While severe AKI after prostatectomy appears to be infrequent, transurethral resection of the prostate and transurethral rhabdomyolysis appear to increase the risk [17]. Propensity score matching analysis found that RARP had better outcomes than retropubic radical prostatectomy in terms of blood loss and hospital length of stay (*p* < 0.001). It also was associated with a much lower incidence of AKI (5.5% versus 10.4%, *p* = 0.044) [18]. Previous studies did not appear to take into account existing CKD. In the present study, patients with CKD had more postoperative AKI and urinary complications than did non-CKD patients. We must also point out that the overall incidence of AKI might be underestimated in the present study. AKI involves small changes in creatinine and urinary output. Identifying AKI using billing codes has low sensitivity, which may result in bias [19]. Grams et al. (2014) [19] compared billing code-identified AKI with the 2012 Kidney Disease Improving Global Outcomes (KDIGO) creatinine-based criteria and an approximation of the 2012 KDIGO creatinine- and urine output- based criteria in a subset with available outpatient data. It has been concluded that the use of billing codes to identify AKI has low sensitivity compared with the current KDIGO consensus definition, especially when the urine output criterion is included, and results in the identification of a more severe phenotype [19].

In the present study, we conducted additional analyses to compare results by type of surgery. A previous study of NIS data from 2001 to 2007 found that patients who received minimally invasive surgery (as compared to open surgery) had a shorter length of stay (OR = 0.61, 95% CI: 0.54–0.69, *p* < 0.001) [20]. Similarly, one study from Japan compared 89 consecutive cases of RARP with 105 cases of open surgery; those receiving RARP had fewer postoperative infections, although the results were not significant [21]. A previous meta-analysis of two single-setting studies (*n* = 446) found slight improvement in certain postoperative outcomes of RARP versus ORP and concluded that this “probably” was associated with reduced length of hospital stay [11]. A 2018 review and meta-analysis of RARP versus ORP in 19 studies (*n* = 16,830) performed around the world found inconsistent results, although RARP was associated with better results in terms of blood loss, transfusion, nerve-sparing, recovery of urinary continence, and recovery of erectile function [22]. In another recent study, Saika et al. (2018) [23] reported the use of RARP in high-risk locally advanced prostate cancer as an option providing optimal outcomes; the included studies reported acceptable perioperative and oncological outcomes as well as improved survival. In addition, advantages of RARP, including tissue magnification and recognition, and tridimensional vision, are shown to aid the recovery of urinary continence [23]. Unlike these previous studies, the present study compared RARP to ORP according to patients’ kidney function. All had lower rates of postoperative complications, and non-CKD and CKD patients had shorter hospital stays. The better results of RARP might also be explained by the reasons mentioned above. However, confounding by indication could not be ruled out given the design of the present study. Thus, readers are advised to interpret these results with caution.

## Limitations

The present study has several limitations. First, patients with CKD or ESRD were identified using ICD-9-CM codes. Although the ICD-9 diagnosis for CKD is highly specific, its sensitivity is only around 80% [24]. Therefore, some patients with milder degrees of CKD may have been misclassified as not having CKD. Comorbidities and postoperative complications were identified using ICD-9 and CCS codes, by which the severity of comorbidities could not be indicated. As mentioned above, the use of billing codes to identify AKI has low sensitivity, which may result in bias. Due to significant overlap between patients, the (non-ESRD) CKD cohort could not be separated into individual groups based on stages, and management of CKD could not be accounted for because information was not included in the database. The NIS database also did not include data on prostate-specific antigen (PSA) levels, Gleason scores and prostate cancer management; complications definitions and management; the surgical course or alternate treatments (e.g., use of contrast agents and non-surgical management options for prostate cancer (e.g., chemotherapy, radiotherapy, or hormone therapy) that may independently contribute to adverse outcomes. In addition, other possible confounding variables not collected by NIS were necessarily excluded from our analyses. Finally, since we did not include follow-up data after discharge, we were unable to evaluate late morbidity and oncological outcomes. Despite these limitations, the NIS data provided a 10-year history of representative US patients undergoing radical prostatectomy and their post-surgical outcomes with or without underlying CKD and ESRD, representing an important strength of the present study and adding credence to the results.

## Conclusion

In conclusion, patients with CKD and ESRD undergoing radical prostatectomy for prostate cancer have a greater likelihood of postoperative morbidities and longer hospital stays than those without CKD. Patients receiving radical prostatectomy should be carefully evaluated for kidney dysfunction, as this factor significantly affects post-surgical outcomes. RARP appears to have better outcomes than ORP in terms of postoperative complications and length of stay; however, these results need to be further confirmed. Further investigation and vigilance in treating the CKD/ESRD population, and evaluating the possible influence of CKD stages, is highly warranted.

## Additional file

Additional file 1: **Table S1.** Multivariate models of clinical outcomes by extent of kidney disease, demographic characteristics, hospital characteristics, and comorbidities (DOCX 23 kb)

## Data Availability

The datasets used and/or analyzed during the current study are available from the corresponding author on reasonable request.

## Abbreviations

AHRQ: Agency for Healthcare Research and Quality
AKI: Acute kidney injury
CCS: Clinical classifications software
CKD: Chronic kidney disease
DVT: Deep vein thrombosis
eGFR: Estimated glomerular filtration rate
ESRD: End-stage renal disease
HCUP: Healthcare cost and utilization project
KDIGO: Kidney disease improving global outcomes
LRP: Laparoscopic radical prostatectomy
MRI: Magnetic resonance imaging
NIS: National Inpatient Sample
ORP: Open radical prostatectomy
PSA: Prostate specific antigen
RARP: Robot-assisted radical prostatectomy;
RRT: Renal replacement therapy
