# Supplementary material for: Post-surgical outcomes of patients with chronic kidney disease and end stage renal disease undergoing radical prostatectomy: 10-year results from the US National Inpatient Sample
Source: BMC Nephrol. 2019 Jul 23;20:278. doi: 10.1186/s12882-019-1455-2 (PMC6651956; doi:10.1186/s12882-019-1455-2)
Supplement: Supplementary file 1 — Table S1. Multivariate models of clinical outcomes by extent of kidney disease, demographic characteristics, hospital characteristics, and comorbidities (DOCX 23 kb) [file 12882_2019_1455_MOESM1_ESM.docx]

**Table S1. Multivariate models of clinical outcomes by extent of kidney disease, demographic characteristicss, hospital characteristics, and comorbidities**

| Variables | Postoperative complications | Acute renal failure‡ | Length of stay |
| --- | --- | --- | --- |
|  | aOR (95% CI) | aOR (95% CI) | β ± SE |
| Non-CKD | Reference | Reference | Reference |
| CKD | **1.36 (1.18-1.56)** | **5.16 (4.32-6.17)** | **0.41 ± 0.10** |
| ESRD | 1.35 (0.97-1.87) | - | **1.12 ± 0.40** |
| **Surgical approach** |  |  |  |
| Robot-assisted | Reference | Reference | Reference |
| Open | **0.54 (0.5-0.59)** | 1.06 (0.94-1.18) | **-0.75 ± 0.03** |
| **Demographic data** |  |  |  |
| Age | **1.01 (1.01-1.02)** | 1.00 (1.00-1.01) | **0.005 ± 0.001** |
| Race |  |  |  |
| Missing | **1.23 (1.07-1.40)** | 1.10 (0.94-1.30) | **0.14 ± 0.04** |
| White | Reference | Reference | Reference |
| Black | **1.19 (1.10-1.29)** | **1.39 (1.20-1.60)** | **0.35 ± 0.03** |
| Hispanic | 1.01 (0.92-1.12) | 0.95 (0.74-1.21) | **0.15 ± 0.04** |
| Other | **1.15 (1.03-1.29)** | 1.01 (0.78-1.29) | **0.18 ± 0.05** |
| Income |  |  |  |
| Missing | **0.86 (0.75-0.98)** | 0.85 (0.59-1.23) | 0.01 ± 0.04 |
| 0-25th percentile | Reference | Reference | Reference |
| 26th to 50th percentile | 0.95 (0.89-1.01) | 0.94 (0.82-1.08) | **-0.08 ± 0.02** |
| 51st to 75th percentile | **0.88 (0.83-0.95)** | 0.91 (0.79-1.05) | **-0.08 ± 0.02** |
| 76th to 100th percentile | **0.83 (0.77-0.90)** | **0.80 (0.69-0.93)** | **-0.16 ± 0.02** |
| Insurance status |  |  |  |
| Missing | 1.21 (0.74-1.97) | 0.60 (0.15-2.31) | -0.15 ± 0.11 |
| Medicare/Medicaid | Reference | Reference | Reference |
| Private/HMO | **0.90 (0.85-0.94)** | 0.89 (0.79-1.01) | 0.06 ± 0.04 |
| Self-pay/no-charge/other | 0.92 (0.82-1.03) | 1.06 (0.82-1.37) | **-0.09 ± 0.02** |
| **Hospital characteristics** |  |  |  |
| Location and teaching status |  |  |  |
| Missing | 1.21 (0.75-1.97) | 1.49 (0.73-3.06) | 0.31 ± 0.22 |
| Rural | Reference | Reference | Reference |
| Urban nonteaching | 1.02 (0.89-1.17) | **1.33 (1.02-1.74)** | -0.04 ± 0.05 |
| Urban teaching | 0.90 (0.78-1.04) | 1.24 (0.95-1.62) | -0.10 ± 0.05 |
| Region |  |  |  |
| Northeast | Reference | Reference | Reference |
| Midwest | 1.14 (1.00-1.30) | **1.34 (1.11-1.61)** | -0.05 ± 0.06 |
| South | 0.94 (0.84-1.05) | 1.03 (0.87-1.22) | **-0.14 ± 0.04** |
| West | 0.98 (0.87-1.11) | 1.05 (0.87-1.27) | **-0.12 ± 0.05** |
| Radical prostatectomy caseload^1^ |  |  |  |
| 0-25th percentile | Reference | Reference | Reference |
| 26th to 50th percentile | **0.84 (0.78-0.91)** | **0.86 (0.76-0.99)** | **-0.21 ± 0.03** |
| 51st to 75th percentile | **0.62 (0.56-0.69)** | 0.93 (0.80-1.09) | **-0.47 ± 0.03** |
| 76th to 100th percentile | **0.47 (0.38-0.57)** | 0.84 (0.70-1.02) | **-0.63 ± 0.05** |
| **Comorbidities** |  |  |  |
| Elixhauser comorbidity score^2^ |  |  |  |
| 0-25th percentile | Reference | Reference | Reference |
| 26th to 50th percentile | **1.32 (1.23-1.41)** | **1.78 (1.47-2.15)** | **0.20 ± 0.03** |
| 51st to 75th percentile | **1.66 (1.51-1.84)** | **2.80 (2.22-3.52)** | **0.42 ± 0.04** |
| 76th to 100th percentile | **1.94 (1.68-2.24)** | **4.05 (2.99-5.49)** | **0.74 ± 0.07** |
| Anemia | 1.09 (0.98-1.21) | **1.96 (1.66-2.31)** | **0.82 ± 0.06** |
| Congestive heart failure | **1.97 (1.65-2.34)** | **2.03 (1.53-2.69)** | **0.92 ± 0.13** |
| Chronic pulmonary disease | 1.02 (0.94-1.10) | 0.87 (0.74-1.02) | -0.05 ± 0.03 |
| Coagulopathy | **3.23 (2.73-3.81)** | **2.07 (1.57-2.74)** | **0.98 ± 0.14** |
| Depression | 1.04 (0.94-1.15) | 0.87 (0.70-1.09) | **-0.17 ± 0.03** |
| Diabetes | **0.90 (0.84-0.97)** | 0.98 (0.86-1.11) | **-0.15 ± 0.03** |
| Hypertension | **0.85 (0.79-0.91)** | 0.88 (0.77-1.01) | **-0.21 ± 0.02** |
| Fluid/electrolyte disorders | **4.93 (4.51-5.38)** | **7.99 (6.94-9.20)** | **1.95 ± 0.09** |
| Obesity | 1.06 (0.97-1.16) | **1.27 (1.09-1.48)** | **-0.14 ± 0.03** |
| Peripheral vascular disorders | 1.02 (0.87-1.20) | 1.05 (0.77-1.43) | -0.02 ± 0.08 |
| Weight loss | **5.44 (3.98-7.44)** | **7.39 (5.03-10.86)** | **8.71 ± 1.00** |

SE: standard error; β: beta-coefficient; OR: odds ratio; aOR: adjusted odds ratio; CI: confidence interval; CKD, chronic kidney disease; ESRD, end stage renal disease; HMO, health maintenance organization.

Significant values are in bold.

Multivariate analyses were adjusted for significant baseline characteristics, including age, race, income, insurance status, hospital location and teaching status, region, radical prostatectomy caseload, and all comorbidities.

‡ The ESRD group was not included in the model of acute renal failure.

^1^ Hospital annual radical prostatectomy caseload was defined using quartiles (Q_1_ = 22, Q_2_ = 69, Q_3_ = 169).

^2^ Elixhauser comorbidity score was defined using quartiles (Q_1_ = 0, Q_2_ = 1, Q_3_ = 2).
